# Future directions in systemic treatment of metastatic hormone-sensitive prostate cancer

**DOI:** 10.1007/s00345-022-04135-8

**Published:** 2022-08-27

**Authors:** Kenneth Chen, Louise Kostos, Arun A. Azad

**Affiliations:** 1 Department of Urology, Singapore General Hospital, Singapore, Singapore; 2 Sir Peter MacCallum Department of Oncology, University of Melbourne, Melbourne, VIC Australia; 3 Department of Medical Oncology, Peter MacCallum Cancer Centre, Melbourne, VIC Australia

**Keywords:** Prostate cancer, Advanced prostate cancer, Androgen pathway receptor inhibitors, Treatment intensification, Combination treatment

## Abstract

The landscape of advanced prostate cancer treatment has evolved tremendously in past decades. The treatment paradigm has shifted from androgen deprivation therapy (ADT) alone to doublet combinations comprising ADT with docetaxel or an androgen receptor inhibitor, and now triplet therapy involving all 3 classes of agents. Robust clinical data has demonstrated survival benefits with this strategy of upfront treatment intensification. Subgroup analysis has alluded to the importance of tailoring treatment according to metastatic disease burden. However, defining the volume of disease is becoming increasingly controversial due to the advent of next generation molecular imaging. Several trials testing established agents in the castrate-resistant setting are now underway in metastatic hormone sensitive prostate cancer patients. As the treatment milieu is enriched earlier in the disease trajectory, future studies should elucidate biomarkers to further define specific patient populations who will benefit most from treatment intensification and/or de-escalation, with what agents and for what duration.

## Current Landscape of metastatic hormone-sensitive prostate cancer

The landscape of advanced prostate cancer treatment has evolved tremendously in the past two decades [1]. Several factors contributed to the rapidly changing paradigm including improved understanding of the tumour biology as well as the development of several novel agents.

Androgen deprivation therapy (ADT) has been the backbone of metastatic prostate cancer treatment since the androgen sensitivity of prostate cancer was established more than eight decades ago. Since the discovery of the Gonadotropin-releasing hormone (GnRH) signalling pathway in 1971 [2], luteinizing hormone-releasing hormone (LHRH) agonist has been the agent of choice in part due to challenges with the production of an effective antagonist alternative. However, antagonists have the distinct advantages of avoiding a testosterone flare, rapid achievement of castration and more importantly, a lower risk of major cardiovascular events [3]. The first oral formulation of a GnRH antagonist was compared to leuprolide depot in a phase 3 randomised controlled trial and demonstrated superiority in the primary endpoint of sustained castration rate and key secondary endpoints such as probability of castration at day 4 and of profound testosterone suppression (< 20 ng per deciliter) [4].

The concept of intensifying treatment by combining a luteinizing hormone-releasing hormone (LHRH) agonist with another agent was explored as early as the 1990s by two phase III studies conducted by The European Organization for Research and Treatment of Cancer (EORTC), albeit with conflicting results [5]. The EORTC GU Group Trial 30,843 compared maximal androgen blockade using a LHRH agonist plus cyproterone acetate versus standard LHRH monotherapy or bilateral orchiectomy and found no differences in survival, response rates and time to progression [6]. In contrast, Dennis et al. reported significantly improved overall survival (OS), time to progression and progression-free survival (PFS) in patients receiving LHRH plus flutamide compared to those with bilateral orchiectomy [7]. Given the paucity of high-level evidence to support intensification of treatment, guideline recommendations for maximal androgen blockade remained weak in the ensuing years.

The interest in treatment intensification for metastatic prostate cancer was reignited in 2015 when the interim analyses from CHAARTED trial reported a 13.6 months increase in median OS for the combination of ADT with docetaxel compared to ADT alone with a PFS of 20.2 months with docetaxel compared with 11.7 months for ADT alone in men with newly diagnosed metastatic hormone-sensitive prostate cancer (mHSPC) [8]. This positive signal from chemohormonal treatment remained clear with updated follow-up data (median OS 57.6 v 47.2 months; HR for death in the combination arm, 0.72; 95% CI, 0.59 to 0.89; *P* = 0.0018), and was shown to be more pronounced for the subgroup of patients with high-volume disease, defined as the presence of visceral metastases or ≥ 4 bone lesions with ≥ 1 beyond the vertebral bodies and pelvis (HR for death in the combination arm, 0.63; 95% CI, 0.50 to 0.79; *P *< 0.001) compared to patients with low-volume disease where an overall survival benefit could not be confirmed (HR 1.04; 95% CI, 0.70 to 1.55; *P* = 0.86) [9]. The STAMPEDE study population differed by including M0 patients along with M1 patients. Nonetheless, it confirmed the OS benefit of upfront combination of docetaxel and ADT (HR = 0.81, 95% CI 0.69–0.95, *P* = 0.009) with no evidence that benefit differed by metastatic burden [10, 11].

The LATITIUDE was a multicenter, randomized, double-blind, phase 3 trial on high-risk de novo mHSPC, defined as men who had at least 2 of 3 high-risk prognostic factors (GS ≥ 8, ≥ 3 lesions on bone scan, and visceral metastases, excluding lymph node metastasis) [12]. The promising results from this trial demonstrating OS benefit with abiraterone acetate and prednisolone in addition to ADT for men with de novo, high risk mHSPC (HR 0.66 CI 0.56–0.78, *p* < 0.0001) heralded the era of next generation androgen receptor pathway inhibitors (ARIs). In the following years, three more ARIs were introduced with convincing OS and PFS benefits that apply to patients regardless of de novo or recurrent disease, and metastatic burden.

Together the CHAARTED and LATITUDE studies were prescient in their design, incorporating unprecedented definitions of high/low-volume and high/low-risk disease as exploratory risk stratification of mHSPC. This provided early parameters to guide selection of patients for intensification of treatment especially with docetaxel, which has been shown to yield a greater benefit in high volume disease. Recently, both criteria have also proven to be prognostic as well at diagnosis of mCRPC, with CHAARTED criteria being independently associated with PFS and OS, while the LATITUDE criteria was an independent prognostic factor for only PFS [13].

ARCHES was a multinational, double-blind, phase III trial, which randomised 1,150 men with mHSPC to enzalutamide (160 mg/day) or placebo, plus ADT. The trial reported an improvement in rPFS [14] (hazard ratio, 0.39; 95% CI, 0.30 to 0.50; *P* < 0.001) and OS [15] (median not reached in either group; hazard ratio, 0.66; 95% CI, 0.53 to 0.81; *P* < 0.001). ENZAMET, another large phase 3 study, compared enzalutamide with another non-steroidal antiandrogen (NSAA) in combination with ADT in mHSPC patients and again reported significant improvement in OS (HR 0.67, 95% CI 0.52–0.86) and other secondary endpoints including time to PSA rise (HR 0.39, 95%CI 0.33–0.47) and time to clinical progression (HR 0.40, 95%CI 0.33–0.49) [16]. The efficacy of apalutamide, another potent next-generation ARI, was investigated in the TITAN study which demonstrated a 35% reduction in risk of death over placebo for men who received apalutamide in addition to ADT (48% reduction after adjusting for crossed over from placebo to apalutamide). There was consistent benefit with apalutamide for time to castration resistance as well as PFS [17]. Darolutamide is the most recent next-generation ARI at the time of this publication and is being investigated in addition to ADT in mHSPC patients in the ongoing double-blind phase 3 ARANOTE trial (NCT04736199).

## Role of treatment intensification

The consistent survival benefit of ARIs have collectively put forth a strong argument for early intensification of treatment. This burgeoning evidence (Table 1) together with the positive result from the CHAARTED trial led to interest in exploring the synergistic effect of the two classes of agents as a combination therapy.

**Table 1:** Landmark trials in mHSPC

Trial	Year 1st published	Agent (comparator)	FDA approval date	OS (HR 95% CI)	PFS equivalent outcome (HR 95% CI)
CHAARTED [8]	2015	DOC + ADT (ADT)	-	0.61 (0.47—0.8) P < 0.001	0.61 (0.50—0.75) P < 0.001
LATITUDE [12]	2017	AAP + ADT (PBO + ADT)	7 February 2018	0.62 (0.51—0.76) P < 0.001	0.47 (0.39—0.55) P < 0.001
ARCHES [14, 15]	2019	ENZ + ADT (PBO + ADT)	16 December 2019	0.66 (0.53—0.81) P < 0.001	0.63 (0.52—0.76) P < 0.001)
ENZAMET [16]	2019	ENZ + ADT (NSAA + ADT)		0.67 (0.52—0.86) P = 0.002	0.39 (0.33—0.47) P < 0.001
TITAN [17]	2019	APA + ADT (PBO + ADT)	17 September 2019	0.67 (0.51—0.89) P = 0.005	0.48 (0.39—0.60) P < 0.001

*FDA* US Food and Drug Administration, *HR* hazard ratio, *OS* overall survival, *PFS* progression-free survival, *DOC* docetaxel, *ADT* androgen deprivation therapy, *AAP* abiraterone acetate + prednisolone, *PBO* placebo, *ENZ* enzalutamide; *NSAA* non-steroidal anti-androgen, *APA* apalutamide

Subgroup analyses of patients in the ARCHES, ENZAMET and TITAN trials who received docetaxel prior to randomization provided the earliest insights into the efficacy of triplet therapy. While ARCHES and TITAN included patients with prior docetaxel, ENZAMET included a mixture of prior and concurrent triple therapy regimens with 159 of the 243 (65%) patients planned for early use of docetaxel in the enzalutamide arm having combination therapy (Table 2). Survival benefit was not established for the combination of docetaxel and ARI in addition to ADT in these trials and it was not until the PEACE-1 trial that OS benefit with triplet therapy was demonstrated for the first time.

**Table 2:** Trials with available data evaluating triplet therapy

Trial	Patients on triplet therapy, n	Percentage of study population	Timing of Docetaxel in relation to ARI	Docetaxel Cycles	Impact on OS HR; 95% CI
ARCHES [61]	205	17.8%	Prior	6 cycles (86%)	0.74 (0.46–1.20)
ENZAMET [16]	159	14.1%	Prior (35%) Concurrent (65%)	6 cycles (71%)^§^	0.90 (0.62–1.31)
TITAN [17]	58	10.7%	Prior	6 cycles (median)	1.12 (0.59–2.12)
PEACE-1 [19]	355^†^	30.3%	Concurrent	6 cycles (100%)	0.75 (0.59–0.95)
ARASENS [20]	651	50%	Concurrent	6 cycles	0.68 (0.57–0.80)

HR, hazard ratio; OS, overall survival; CI, confidence interval

^†^Number of patients randomized to abiraterone

^§^Percentage of the subset of patients who had planned early use of docetaxel in ENZAMET

The prospective randomized phase III PEACE-1 trial investigated the additive benefits of abiraterone compared to ADT with docetaxel alone in men with de novo hormone-sensitive metastatic prostate cancer [18]. Docetaxel was not included in the original design of the study, however, was permitted as part of standard of care (SOC) treatment in 2015 and made compulsory from 2017 onwards following report of the CHAARTED trial results which established upfront docetaxel as evidence-based treatment for patients with newly diagnosed metastatic prostate cancer. Benefit of the triplet combination was demonstrated for co-primary endpoints with a median of 2.3 years advantage in radiographic PFS (HR 0.54, 95% CI 0.46–0.64, *p* < 0.0001) and significantly improved OS (HR 0.75, 95% CI 0.59–0.95, *p* = 0.017). The survival benefit was restricted to patients with high-volume disease (HR 0.72, 95% CI 0.55–0.95, *p* = 0.019), with the effect not seen in low-volume disease (HR 0.83, 95% CI 0.5–1.38, *p* = 0.66) [19].

ARASENS is a randomised phase 3 trial investigating the triplet combination of darolutamide with docetaxel and ADT versus docetaxel and ADT alone, the results of which were recently reported [20]. In contrast to PEACE-1 which only investigated men with de novo mHSPC, ARASENS included men with recurrent disease (13.9%) as well. The study showed that triplet combination significantly reduced the risk of death by 32.5% (HR 0.68, 95% CI 0.57–0.8, *p* < 0.001) and this benefit was largely consistent across pre-specified subgroups although metastatic burden was not subanalysed and little can be said confidently about the benefit in patients with recurrent disease due to poor representation in the study. There was improvement with triplet therapy in secondary endpoints of time to castration-resistance (HR 0.36, 95% CI 0.30–0.42, *p* < 0.001) and time to pain progression (HR 0.79, 95% CI 0.66–0.95, *p* = 0.01). The rates of adverse events were similar in both arms (44.8% vs 42.3%).

Of note, ARASENS and PEACE-1 (following an amendment) are the only randomised trials whereby docetaxel was mandated in all patients. This is in contrast to the ARCHES, ENZAMET and TITAN trials where docetaxel was given at physician’s discretion. This potentially introduces confounders that may impact on outcomes since younger/fitter patients and those with more aggressive disease characteristics are more likely to be offered docetaxel.

However, treatment intensification is not a one-way treatment philosophy. A balanced approach must tackle pertinent issues such as duration of intensification and also which patients to de-escalate treatment. Drawing lessons from the evidence on intermittent ADT [20, 21], the feasibility of intermittent regimens should be considered after an initial period of treatment intensification in selected patients. Biomarkers are needed to guide patient selection for treatment de-escalation. Further trials are needed to define the optimal strategy of treatment de-escalation, including which agent to de-escalate and when to de-escalate.

## The role of radiotherapy in treatment intensification of mHSPC

While the strategy of treatment intensification has been defined thus far by combination of pharmacological agents, it can also be viewed as a multimodality treatment where systemic therapy is combined with local treatment of the primary tumour or metastases-directed therapy (MDT). Radiotherapy (RT) has proven benefits in this setting.

Treatment of the primary tumour in de novo mHSPC.

The HORRAD trial investigated the benefit of prostate radiotherapy in 432 patients randomized to ADT alone or ADT plus Intensity-modulated RT to the prostate. There was no OS benefit (HR: 0.9 [0.7–1.14]), although median time to PSA progression was significantly improved in the RT arm (HR: 0.78 [0.63–0.97]) [22]. The STAMPEDE trial similarly showed no survival benefit in unselected patients. However, a clear overall and biochemical recurrence-free survival exists for the low-volume (CHAARTED criteria) subgroup [23]. This was further confirmed in a meta-analysis of both trials [24], thus defining the standard of care for patients with low volume synchronous metastases.

### Metastasis-directed therapy for recurrent oligometastatic disease

Beyond advancements in systemic therapy, metastases-directed therapy is another strategy that is beginning to refine the management of mHSPC. The concept hinges on the understanding of metastasis as a disease spectrum rather than a binary state of all-or-nothing. This dogma was challenged as early as the 1990s with the recognition of a low-volume metastatic state otherwise known as oligometastasis. With the burgeoning use of PSMA PET/CT, the opportunity to improve outcomes by consolidation of low volume disease is now ever more appealing. Two recent phase 2 RCTs provided early insights into the benefits of MDT in oligometastatic prostate cancer.

The ORIOLE study [25] compared observation versus SABR in 54 patients with recurrent hormone-sensitive prostate cancer and 1–3 metastases detected by conventional imaging. The study showed an improvement in median PFS with SABR (not reached vs 5.8 months; hazard ratio, 0.30; 95% CI, 0.11–0.81; *P* = 0.002). The STOMP trial [26] randomised patients with hormone-sensitive oligorecurrent disease in 1:1 ratio to surveillance or metastasis directed therapy. In the 62 men enrolled, the median ADT-free survival was improved in those with MDT (21 vs 13 months, hazard ratio, 0.60 [80% CI, 0.40 to 0.90]; *P* = 0.11). Other prospective trials investigating MDT in the oligorecurrent disease setting include the POPSTAR trial [27], assessing a single fraction of 20-Gy SABR to each oligometastasis, and the TRANSFORM trial [28], which examines fractionated SBRT (50 Gray in 10 fractions) to each metastatic lesion. Both showed very similar benefits in terms of delay of treatment escalation at 2 years (48% and 51.7%, respectively).

Pooled data suggest metastasis-directed therapy improves both biochemical-free (HR: 0.44, 95%CI 0.29–0.67, *p* < 0.01) and ADT-free survival (HR: 0.56, 95%CI 0.34–0.94, *p* = 0.03) as compared to active surveillance with low rates of grade 3 or higher toxicity [29]. Nonetheless, large trials are needed to further establish its benefits. Ongoing trials studying MDT in the hormone-sensitive oligorecurrent space include the PEACE V STORM (NCT03569241), PLATON trial (NCT03784755), RAVENS (NCT04037358) and ECOG-ACRIN 8191 (NCT04423211).

## Next generation imaging and its impact on the management of mHSPC

The advent of a more sensitive imaging modality can lead to the Will Roger’s phenomenon. This stage migration is seen in prostate cancer where a proportion of high-risk localized prostate cancer patients that are negative with conventional imaging but demonstrate metastases on PSMA PET/CT are reclassified to the oligometastatic cohort of patients. This shift invariably improves outcomes of the group with localised disease by removing those that are truly oligometastatic, and at the same time also improves outcomes of the metastatic patients by introducing low volume oligometastatic patients to this group.

It seems likely that with more widespread use of PSMA PET for staging unfavourable-intermediate and high-risk localised prostate cancer based on the ProPSMA trial data [30], that the incidence of mHSPC will increase. This poses a key clinical dilemma as to how to best manage patients with localised disease on conventional imaging but evidence of metastatic disease on PSMA PET. For example, should these patients be offered local therapy ± metastasis-directed therapy or should systemic therapy be the main treatment modality? Until PSMA PET is widely incorporated into prospective clinical trials, these dilemmas will persist.

PSMA PET also has implications when considering the options available for patients with de novo low volume vs high volume disease. Radiotherapy to the primary tumour for patients with low metastatic burden (by CHAARTED criteria) has now become the standard of care [24, 31, 32]. It remains unknown if this treatment strategy should still apply to the eligible patient with oligometastases on conventional imaging who is now reclassified by PSMA PET/CT as high volume disease. Similarly, whether these patients upstaged to high volume disease PSMA PET/CT should receive docetaxel is a point of contention.

## New therapeutic agents and current trials in mHSPC

Most novel agents discussed hitherto were evaluated initially in the metastatic castrate-resistant prostate cancer (mCRPC) setting. With the emphasis on utilising therapies earlier in the prostate cancer treatment paradigm to intensify upfront treatment, several other novel agents currently approved for use in the mCRPC space are also under evaluation in the mHSPC setting [33, 34].

^177^Lu-PSMA, which has recently been FDA approved for use in mCRPC, is currently being studied in patients with locally advanced and mHSPC. The UpFrontPSMA trial is a randomised phase II study comparing the combination of ^177^Lu-PSMA-617 with upfront docetaxel chemotherapy to docetaxel alone in patients with de novo high volume mHSPC [35]. The rationale behind this study is that the addition of ^177^Lu-PSMA will hopefully lead to higher disease eradication and, therefore, prolong the time to developing castration resistance. Recruitment is currently ongoing. Similarly, the PSMAddition trial randomises patients with mHSPC to receive standard treatment with ADT and an ARI, either with or without ^177^Lu-PSMA-617 [36]. Depending on whether these trials meet their primary endpoints and demonstrate superior efficacy in the ^177^Lu-PSMA cohorts, the use of ^177^Lu-PSMA may eventually be integrated into standard upfront treatment for mHSPC.

Similarly, following the publication of the PROfound study results demonstrating a survival benefit with the PARP inhibitor olaparib in BRCA-mutated mCRPC, the FDA approved its use in mCRPC after progression on an ARI [37]. The National Comprehensive Cancer Network (NCCN) currently recommends somatic and germline testing for HRR mutations (such as BRCA1 and BRCA 2) in all patients with metastatic prostate cancer [38]. PARP inhibitors are currently being evaluated in the mHSPC space (see Table 3), alone and with ARIs. Both preclinical and clinical data suggest that combining AR and PARP inhibitors may be synergistic and delay disease progression when used in hormone-sensitive disease. Talazoparib, a potent PARP inhibitor, is being evaluated in patients with mHSPC in combination with abiraterone and ADT (NCT04734730) as well as enzalutamide and ADT (TALAPRO-3, NCT04821622). Niraparib, a selective inhibitor of PARP1 and PARP2, is also being evaluated in the AMPLITUDE trial where it is given in combination with abiraterone and ADT (NCT04497844). The latter two studies require the presence of a somatic or germline HRD aberration. Several other studies are currently ongoing assessing the benefit of a PARP inhibitor in mHSPC (see Table 3).

**Table 3:** Current trials evaluating novel therapies and treatment combinations in patients with mHSPC

Trial	Experimental intervention	Disease group	Phase	Primary outcome
^177^Lu-PSMA				
UpfrontPSMA (NCT04343885)	LuPSMA + Docetaxel	De novo, high volume mHSPC	II	Undetectable PSA at 12 months
PSMAaddition (NCT04720157)	LuPSMA + ARI + ADT	mHSPC	III	rPFS
Bullseye (NCT04443062)	LuPSMA	Relapsed oligometastatic mHSPC	II	Proportion of patients with disease progression within 6 months
NCT05079698	LuPSMA + SABR	Relapsed oligometastatic mHSPC	I	Proportion of patients with DLT
PARP inhibitors				
FAALCON (NCT04748042)	Olaparib + Abiraterone + ADT + SABR	Relapsed oligometastatic mHSPC	II	Percentage of patients without treatment failure at 24 months
NCT05167175	Olaparib + Abiraterone	HRD mutant mHSPC	II	rPFS
TRIUMPH (NCT03413995)	Rucaparib	Germline HRD mutant mHSPC	II	PSA-RR
NCT04734730	Talazoparib + Abiraterone + ADT	mHSPC	II	PSA nadir < 0.2 at 12 months
ZZ-first (NCT04332744)	Talazoparib + Enzalutamide	High volume mHSPC	II	PSA-complete response
TALAPRO-3 (NCT04821622)	Talazoparib + Enzalutamide	DDR-mutated mHSPC	III	rPFS
AMPLITUDE (NCT04497844)	Niraparib + Abiraterone	HRD mutant mHSPC	III	rPFS
MAGNITUDE (NCT03748641)	Niraparib + Abiraterone	HRD mutant + wild-type mHSPC	III	rPFS
AKT inhibitors				
CAPItello281 (NCT04493853)	Capivasertib + Abiraterone	De novo mHSPC, PTEN deficiency confirmed on IHC	III	rPFS
CDK4/6 inhibitors				
CYCLONE-3 (NCT05288166)	Abemaciclib + Abiraterone	High-risk mHSPC	III	rPFS
PD-1/PD-L1 inhibitors				
CABIOS (NCT04477512)	Nivolumab + Cabozantinib + Abiraterone	mHSPC	I	Frequency of DLTs
NCT04262154	Atezolizumab + Abiraterone + ADT + SABR	De novo mHSPC	II	Failure-free rate at 2 years
MAGIC-8 (NCT03689699)	Nivolumab + ADT + BMS986253	Relapsed low-volume mHSPC	I/II	Rate of PSA recurrence, safety and tolerability
POSTCARD (NCT03795207)	Durvalumab + SABR	Relapsed low-volume mHSPC (visible on PET scan only)	II	2 years PFS
NCT03007732	Pembrolizumab + intratumoral SD-101 + Abiraterone + SABR + ADT	Oligometastatic HSPC	II	Number of TRAEs, the rate of PSA < nadir + 2 ng/ml at 15 months
NCT03951831	Cemiplimab + ADT + Docetaxel	mHSPC	II	Percentage of patients with undetectable PSA at 6 months
PROSTRATEGY (NCT03879122)	Nivolumab + Docetaxel + ADT, or Ipilimumab + Nivolumab + Docetaxel + ADT	mHSPC	II/III	OS
NCT04126070	Nivolumab + Docetaxel + ADT	mHSPC	II	Proportion of patients with PSA < 0.2 at 7 months
KEYNOTE-991 (NCT04191096)	Pembrolizumab + Enzalutamide + ADT	mHSPC	III	rPFS, OS
NCT02020070	Ipilimumab + ADT ± prostatectomy	Oligometastatic HSPC	II	Undetectable PSA
Novel vaccines				
UV1/hTERT2012P (NCT01784913)	UV1 vaccine + GM-CSF	Oligometastatic mHSPC	I/II	Safety and tolerability
Novel anti-androgen therapies				
S1216 (NCT01809691)	Orteronel + ADT	mHSPC	III	OS
NCT03520478	SHR3680	HSPC	III	rPFS, OS
NCT04995042	SHR720	HSPC	I	MTD, frequency of DLTs, RP2D

The PI3K-Akt-mTOR pathway has been implicated in prostate cancer development and progression and is deregulated in up to 50% of prostate cancers. Most commonly, this is driven by PTEN loss of function. Prostate cancers with PTEN loss are more sensitive to AKT inhibition, and therefore, PTEN loss can be used as a predictive factor for response to such therapy. The IPATential150 trial evaluated the addition of the AKT inhibitor ipatasertib with abiraterone compared to abiraterone alone [39]. This study demonstrated that in patients with PTEN loss on tumour IHC, the addition of ipatasertib was associated with a PFS benefit [40]. Studies are currently underway evaluating AKT-inhibitors in the hormone-sensitive setting (see Table 3).

The androgen receptor (AR) plays a crucial role in regulating the cell cycle and, therefore, prostate cancer progression towards castration resistance. In particular, it increases cyclin D1, which activates CDK4 and CDK6, allowing the cell to progress from the G1 to S phase. CDK4/6 inhibitors have been used in advanced prostate cancer to disrupt such AR signalling pathways and prevent cell proliferation. The CYCLONE-2 trial (NCT03706365) is currently evaluating whether abemaciclib plus abiraterone is superior to abiraterone alone in mCRPC. A similar trial (CYCLONE-3, NCT05288166) is currently underway in the mHSPC setting. Other novel hormonal agents such as orteronel, a nonsteroidal 17,20-lyase inhibitor (an enzyme necessary for androgen synthesis), are also being evaluated in the mHSPC setting (NCT01809691).

Checkpoint inhibitors have demonstrated limited clinical benefit when used alone in advanced prostate cancer [41, 42]. Prostate cancer has minimal T-cell infiltrates and, therefore, fails to generate a significant peripheral anti-tumour response [43]. In combination with other therapies, checkpoint inhibitors may have a role to play in the management of prostate cancer. One example is through combination with radiotherapy, whereby the radiation is thought to be immunogenic through inducing cytotoxic cell death. The Phase II ICEPAC trial assessed patients with mCRPC treated with avelumab combined with stereotactic radiotherapy and demonstrated an objective response rate of 33% [44]. More extensive studies are warranted to determine whether this translates to a survival benefit. In the mHSPC setting, several similar trials are currently underway combining checkpoint inhibitors with radiotherapy, ARIs or other novel agents (see Table 3).

## Role of biomarkers in the future management of mHSPC

Several emerging biomarkers are under evaluation in mHSPC to aid in risk-stratification of patients, thereby providing a framework to guide initial treatment selection and when to intensify or de-intensify treatment. In addition, such biomarkers can monitor treatment response and predict and detect resistance. In the era of precision medicine, they can also be used to personalise treatment and administer targeted therapies. These biomarkers can be predictive or prognostic and are derived from molecular imaging, tissue or blood samples.

Biomarkers that can be assessed at the initial diagnosis of mHSPC can provide prognostic information about OS, which may assist with patient discussions and choice of therapy. Elevated markers of bone formation (such as C-terminal of type-1 collagen propeptide (CICP) and bone-specific alkaline phosphatase (BAP)), as well as bone resorption markers (N-telopeptide (NTx) and pyridinoline (PYD)), have previously been associated with poor OS outcomes in mCRPC [45]. More recently, patients with mHSPC were prospectively evaluated in the SWOG S1216 trial with bone metabolic biomarkers (CICP, BAP, PYD, and C-telopeptide) (NCT01809691) [46]. An updated analysis of 949 patients found that elevated bone biomarkers at baseline in mHSPC are strongly prognostic for poor OS [47]. Similarly, an elevated platelet-to-lymphocyte ratio (PLR) correlates with a poor disease-free survival (DFS) as well as OS, as demonstrated in a recent meta-analysis [48]. Such markers can aid in initial treatment discussions, particular with regards to escalating systemic therapy and favouring a more aggressive approach. Other markers measured throughout treatment can provide an insight into depth of response and likely outcomes. The PSA level at seven months following commencement of ADT for mHSPC is predictive of survival [49]. Patients with a PSA level of ≤ 0.2 ng/ml at this timepoint have more prolonged survival than those with a higher level, regardless of whether the patient received additional docetaxel.

Another surrogate for biological tumour aggressiveness, in addition to patient factors such as having de novo or high-volume metastatic disease, is the presence and number of circulating tumour cells (CTCs). CTCs can be measured peripherally through a ‘liquid biopsy’ and can assist with predicting response to treatment and guiding whether initial treatment should be intensified. Two prospective studies analysed patients with mHSPC who had samples taken for CTC analysis before starting ADT ± additional systemic therapy. CTC expression at baseline is more likely in patients with high-volume disease (as per CHAARTED criteria [8]) and is predictive of PSA non-response at seven months (PSA > 4.0 ng/ml or progression) [50, 51].

Comprehensive genomic profiling of each patient with mHSPC through liquid biopsy may also play a key role in risk stratification and longitudinal disease monitoring. Circulating tumour DNA (ctDNA) has been extensively studied in mCRPC, however, research is limited in hormone-sensitive disease. Peripheral ctDNA samples have been correlated with baseline and on-treatment biopsies, and in fact represent broader and more complex subclonal populations that genomic analysis of an individual metastatic tissue sample [52]. In a large prospective cohort study evaluating ctDNA in various cohorts of men with prostate cancer, it was found that a higher ctDNA fraction was associated with a shorter time to ADT failure in mHSPC [53]. The ctDNA fraction combined with volume of disease and serum alkaline phosphatase (ALP) levels was also more prognostic of survival than clinical factors alone in mHSPC. In a retrospective study evaluating patients with mHSPC who underwent ctDNA genomic profiling, patients with loss-of-function alterations in Tp53, PTEN or RB1 had shorter PFS overall than the cohort without detectable tumour suppressor genes [54]. On subgroup analysis, the presence of a tumour suppressor gene alteration was also associated with worse outcomes with upfront ADT in combination with abiraterone. Recent data from the phase III TITAN trial reported similar findings, with the presence of any AR aberration in addition to detectable ctDNA at baseline associated with poor OS [55]. The presence of such genomic aberrations could assist with the choice of initial therapy and guide whether to intensify upfront treatment. As discussed above, trials are currently underway evaluating novel therapies for patients with known tumour suppressor gene alterations and mHSPC (see Table 3).

Conversely, an SPOP mutation, which occurs in about 5% of mHSPC patients, is predictive of a favourable response to AR targeted therapies and improved survival outcomes [56]. The presence of an SPOP mutation could influence the choice of initial treatment. Other biomarkers which can assist with initial treatment choice include AR splice variants such as AR-V7. AR-V7 is the most commonly evaluated splice variant and is associated with poor response to ARIs such as enzalutamide or abiraterone in mCRPC. More recently, the presence of AR-V7 on IHC in patients with treatment-naïve mHSPC has also been shown to be predictive of a shorter PFS and lower PSA response rate [57].

Aberrations in the DNA damage repair (DDR) pathway are common in prostate cancer. They are associated with a higher histological grade and a higher chance of developing de novo metastatic disease [58]. In the phase III PROfound trial [37], 2792 patients underwent screening for 15 different DDR aberrations involved in the homologous recombination repair (HRR) pathway. A similar number of aberrations were found in the primary tumour samples compared to the metastatic site samples (27% vs 32%, respectively), suggesting that such alterations occur early in the disease course rather than being acquired as the disease progresses. For mHSPC patients, retrospective data suggest that the presence of germline DDR alterations are predictive of a shorter time for the development of castration-resistant disease [53, 59].

Several imaging biomarkers are already in use in the mCRPC setting. An SUVmean ≥ 10 on PSMA PET is predictive of a favourable response to PSMA-radionuclide based therapy [60]. Conversely, a high metabolic volume of disease on FDG PET scan is prognostic of worse outcomes, specifically rPFS and PSA response rate. Several studies discussed above are currently underway evaluating ^177^Lu-PSMA earlier in the treatment paradigm, including in the mHSPC setting [35]. If these trials are positive and ^177^Lu-PSMA becomes is integrated into the initial standard treatment for mHSPC, next generation PET imaging will become essential in guiding care.

## Conclusion

The evolution of metastatic prostate cancer management in the past decade has been rapid and fast-paced with the approval of several novel agents supported by landmark phase 3 studies, as well as the paradigm shift towards upfront treatment intensification earlier in the disease trajectory. Patients are currently faced with a barrage of treatment options which looks set only to expand in the coming years with newer class agents being added to the current chemohormonal milieu as well as the emerging field of MDT. The development and discovery of biomarkers will undoubtedly help further refine and personalise the metastatic prostate cancer patient’s treatment journey, hopefully with the continued lengthening of meaningful survival years too.
